# Exploring fertility information needs and preferences in young women diagnosed with breast cancer: a qualitative study

**DOI:** 10.1192/j.eurpsy.2023.783

**Published:** 2023-07-19

**Authors:** M. S. Teixeira, A. Bártolo, S. Monteiro

**Affiliations:** 1 Department of Education and Psychology, University of Aveiro, Aveiro; 2 CINTESIS@RISE, Piaget Institute - ISEIT/Viseu, Viseu; 3 CINTESIS@RISE, Department of Education and Psychology, University of Aveiro, Aveiro; 4 Department of Social Sciences and Management, Universidade Aberta, Lisbon, Portugal

## Abstract

**Introduction:**

Research has suggested an increased incidence of breast cancer in young women who have not yet completed family-building projects. However, the use of adjuvant therapies with cytotoxic drugs may affect fertility permanently or transiently. Furthermore, women undergoing prolonged adjuvant hormonal therapy have an increased risk of infertility due to the natural aging of the reproductive system during this period. Thus, young breast cancer survivors present fertility and childbearing concerns and related information needs.

**Objectives:**

The present study aimed to know the experiences of breast cancer survivors regarding the information received about reproductive health during cancer diagnosis and treatment and to identify unmet needs and preferences about how and when to receive this information.

**Methods:**

A exploratory qualitative study was conducted using a convenience sample consisting of young women diagnosed with breast cancer aged 18 to 45 years. Semi‐structured interviews were carried out individually and online with 24 female Portuguese breast cancer survivors (M=37.21; DP=4.44) between June and August 2022.

**Results:**

From the preliminary thematic analysis of the data, three main themes were identified: 1) information received at the time of diagnosis; 2) unmet information needs, and 3) main preferences. Findings showed that most participants received information related to the impact of treatments on fertility, namely about the gonadotoxic effect of chemotherapy. This information was mostly provided by the nurses, but gaps were still identified. The interviews highlighted that, for most participants, it would be important to receive reproductive health information at an early stage of diagnosis, before treatment begins. Breast cancer survivors addressed the need to build a “uniform information model”, as well as booklets that systematize the reproductive impacts of cancer diseases, taking into account the specificities of each type of cancer and associated therapies.

**Conclusions:**

Despite clear indications that fertility is an important issue in the context of breast cancer, the preliminary results of this study suggested that fertility counseling after diagnosis is still limited. There is a need to develop structured interventions that address the reproductive needs and concerns of these patients throughout the course of the disease.

**Disclosure of Interest:**

None Declared

